# WHO International Standard for evaluation of the antibody response to COVID-19 vaccines: call for urgent action by the scientific community

**DOI:** 10.1016/S2666-5247(21)00266-4

**Published:** 2022-03

**Authors:** Ivana Knezevic, Giada Mattiuzzo, Mark Page, Philip Minor, Elwyn Griffiths, Micha Nuebling, Vasee Moorthy

**Affiliations:** a Department of Health Products Policy and Standards, Access to Medicines and Health Products, World Health Organization, Geneva, Switzerland; b Research for Health Department, Science Division, World Health Organization, Geneva, Switzerland; c National Institute for Biological Standards and Control, Potters Bar, UK; d St Albans, UK; e Kingston upon Thames, UK; f Paul-Ehrlich-Institut, Langen, Germany

## Abstract

The first WHO International Standard and International Reference Panel for anti-SARS-CoV-2 immunoglobulin were established by the WHO Expert Committee on Biological Standardization in December, 2020. The WHO International Antibody Standards are intended to serve as global reference reagents, against which national reference preparations or secondary standards can be calibrated. Calibration will facilitate comparison of results of assays (eg, of the neutralising antibody response to candidate COVID-19 vaccines) conducted in different countries. Use of these standards is expected to contribute to better understanding of the immune response, and particularly of the correlates of protection. This Personal View provides some technical details of the WHO Antibody Standards for SARS-CoV-2, focusing specifically on the use of these standards for the evaluation of the immune response to COVID-19 vaccines, rather than other applications (eg, diagnostic or therapeutic). The explanation with regard to why rapid adoption of the standards is crucial is also included, as well as how funders, journals, regulators, and ethics committees could drive adoption in the interest of public health.

## Introduction

Developing, licensing, and rolling out vaccines against an emerging pathogen that is declared a global public health emergency presents many challenges, including accelerated time frames for evaluating safety and efficacy of candidate vaccines. Regulatory processes must be in place for rapid evaluation of submissions and, after careful benefit–risk assessment, the use of vaccines for which a full regulatory package might not yet be available—as in the COVID-19 pandemic declared in 2020.

A broad range of COVID-19 candidate vaccines are being developed via technologies and platforms that include viral vectors, protein subunits, nucleic acids, live attenuated strains, and inactivated virus preparations.^1^, ^2^

One of the important elements of regulatory evaluation is the measurement of the vaccine-induced immune response, for which guidance is available in the WHO guidelines on the clinical evaluation of vaccines.^3^ Evaluation of the immunogenicity of a vaccine aims to identify a possible correlate of protection. Although it is still unclear what such a correlate could be for SARS-CoV-2, neutralising or binding antibodies are good candidates. However, identification of a correlate of protection requires the comparison of immunological data from different clinical trials, but is often confounded by the differences in assays, target antigens, numerical readouts, and endpoints.

This Personal View aims to raise awareness of the need for standardisation of quantitative assays for the evaluation of clinical trials of vaccines intended for use in public health emergencies. The first step towards standardisation is the availability of a WHO International Standard to serve as a reference point for several assays of defined specificity.^4^, ^5^ In May, 2020, a research reagent for anti-SARS-CoV-2 antibody was made available as an interim solution while a collaborative study with the candidates for the International Standard was conducted. A WHO International Standard for neutralising activity of anti-SARS-CoV-2 immunoglobulin has been available since December, 2020, but its usefulness in enabling comparability between vaccines, between laboratories, and over time can be realised only if the International Standard is used widely. To advance standardisation and comparability, it is important that using the WHO International Standard units becomes the norm in the reporting of serological assays for quantitation of the antibody response.^6^

## WHO standards for evaluation of quality, safety, and efficacy of vaccines

WHO has a unique role of supporting regulatory authorities in its 194 Member States. One of WHO's core functions is to set norms and standards and promote and monitor their implementation. The WHO Expert Committee on Biological Standardization has been active in establishing WHO standards for biologicals for over 70 years.

WHO standards, both written and measurement (ie, physical), are based on scientific evidence and provide the basis for establishing and updating national regulatory requirements. Their development is supported by WHO collaborating centres, national regulatory authorities in many countries, pharmacopoeias, manufacturers' associations, and academia. The role of the international recommendations or guidelines for biological substances is to ensure the availability of vaccines of assured quality, safety, and efficacy for use in international immunisation programmes. Furthermore, these documents serve as a benchmark for the global acceptability of products and as a basis for defining national regulatory requirements for licensing and for post-licensure evaluation.

**Figure 1:**
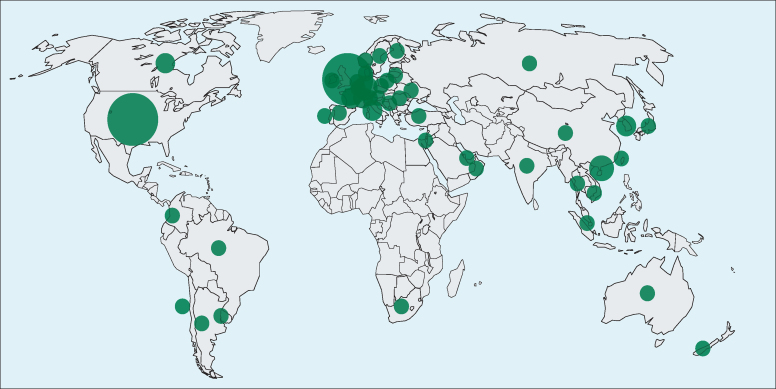
Distribution of the WHO International Standard for anti-SARS-CoV-2 immunoglobulin The map shows the geographical distribution of end users who have acquired the WHO International Standard (National Institute for Biological Standards and Control code 20/136) from December, 2020, until July, 2021. The size of the circle corresponds to the relative number of units shipped per country. The location of the circle within a country is arbitrary and is not pinned to the end user. Overall, more than 2400 units were shipped to 581 individual customers in 46 different countries worldwide.

The development of measurement standards involves elaborate collaborative studies in numerous laboratories worldwide. As examples of the measurement standards for COVID-19, the first WHO International Standard for anti-SARS-CoV-2 immunoglobulin with an assigned unitage of 250 International Units (IU) per ampoule (neutralising antibody activity) and the first WHO International Reference Panel for anti-SARS-CoV-2 immunoglobulin were established by the WHO Expert Committee on Biological Standardization on Dec 10, 2020.^7^, ^8^ Through the WHO Collaborating Centre, the National Institute for Biological Standards and Control, these standards were made available on Dec 18, 2020, under code 20/136 for the WHO International Standard and under the code 20/268 for the Reference Panel.^9^ Figure 1 shows the geographical distribution of the standards to the users worldwide. These standards are intended to serve as global reference reagents against which national reference preparations and commercially available secondary reagents for neutralising antibody activity can be calibrated. The calibration of national references against a single global standard is expected to facilitate comparison of quantitative results of assays (eg, of the neutralising antibody response to candidate COVID-19 vaccines) in different countries.

WHO initiatives are also closely linked to the standardisation of vaccines. Strengthening of national regulatory authorities is an important element in assuring the quality of vaccines worldwide. Prequalification of vaccines is a key mechanism through which WHO ensures the safety and efficacy of vaccines to be supplied by UNICEF. The safety of vaccines and the issues discussed by the WHO Global Advisory Committee on Vaccine Safety are also important, as are WHO activities related to immunisation policy led by the Strategic Advisory Group of Experts on Immunization.

## Scientific basis for developing antibody standards for evaluation of the immune response to COVID-19 vaccines

In 2019–20, a new type of coronavirus was isolated from human respiratory epithelial cells.^10^ The virus, SARS-CoV-2, is the seventh coronavirus discovered that can infect humans.^11^, ^12^, ^13^ Lack of a vaccine, high infectivity, and pathogenicity has led to the classification of SARS-CoV-2 as a hazard group 3 pathogen.^14^, ^15^, ^16^, ^17^ This classification limits accessibility to, and handling of, the live virus for the development and evaluation of antiviral measures to those groups with biosafety level 3 facilities.^18^ Consequently, alternatives to neutralisation assays with the wild-type virus have been developed. These alternatives include various forms of ELISA and surrogates for virus neutralisation assays, such as pseudotyped viruses in which the Spike (S) protein is expressed on the surface of a lentivirus, vesicular stomatitis virus, or another virus.

The S glycoprotein of SARS-CoV-2 is responsible for the virus attachment and entry to the target cells, initiating the infection process while antibodies against it neutralise infectivity. Immune responses against the S protein are key to host protection during SARS-CoV-2 infection, and it is therefore considered the most attractive target for vaccine and therapeutic development.^19^, ^20^, ^21^ The S protein is presented on the virus as a trimer comprising two sections—the S1 and S2 regions. The S1 region includes a sequence of 250–300 amino acids forming the receptor-binding domain that binds to angiotensin-converting enzyme 2 on the target cell, whereas the S2 region serves to fuse the viral and cellular membranes after viral attachment. ELISA formats have included targets such as the whole S protein (which might be modified to stabilise the protein), the S1 protein, the receptor-binding domain, and whole inactivated virus. Assays based on the S2 or the viral nucleoprotein have been used as diagnostic tools to confirm infection.

## Difficulties in comparing immune responses induced by COVID-19 vaccines in clinical trials

Several groups are conducting, or have completed, clinical trials to evaluate the efficacy of their candidate vaccines against SARS-CoV-2; comparison of results between studies, or even between different assays in the same study, has proven difficult. Table 1 summarises three clinical trials of adenovirus-vectored vaccine candidates that were published in 2020.^22^, ^23^, ^24^ These vectors, derived from adenoviruses rendered replication-incompetent by deletion of some early genes and replacement with the S gene of SARS-CoV-2, lead to expression of the S protein when the construct is administered to the individual.

**Table 1:** Immune responses from phase 1 and 2 clinical trials using adenovirus-vectored candidate vaccines

	**ELISA assay**	**ELISA assay**	**ELISA assay**	**Neutralisation assay**	**Neutralisation assay**	**Neutralisation assay**	**Neutralisation assay**
**Jenner Institute ChAdOx1 nCoV-19**^22^							
Output	ELISA unit	ELISA unit	PRNT_50_	PRNT_50_	MN_80_	100% VN	100% VN
Timepoint, days	28	56	28	*..*	28	*..*	56
Titre	157	119*; 639†	218	*..*	51	*..*	29
Proportion of participants with seroconversion (%)	100%	100%	100%	*..*	91%	*..*	62%*; 100%†
**Beijing Institute of Biotechnology-CanSino Biologics**^23^							
Output	1/dil factor	1/dil factor	1/dil factor	1/dil factor	1/dil factor	1/dil factor	1/dil factor
Timepoint, days	28	..	28	*..*	*..*	*..*	*..*
Titre	656·5‡; 571·0 §	..	19·5‡; 18·3§	*..*	*..*	*..*	*..*
Proportion of participants with seroconversion (%)	96%‡; 97%§	..	59%‡; 47%§	*..*	*..*	*..*	*..*
**Gamaleya Institute**^24^							
Output	1/dil factor	1/dil factor	1/dil factor	1/dil factor	1/dil factor	1/dil factor	1/dil factor
Time point, days	28	42	*..*	42	*..*	*..*	*..*
Titre	5382¶; 5322‖	14 703¶; 11 143‖	*..*	49·25¶; 45·95‖	*..*	*..*	*..*
Proportion of participants with seroconversion (%)	100%	100%	*..*	100%	*..*	*..*	*..*

MN_80_=80% microneutralisation. PRNT_50_=50% plaque reduction neutralisation assay. VN=virus neutralisation. 1/dil factor=inverse of the highest dilution factor still positive.

* Single dose.

† Two doses.

‡ High dose.

§ Low dose.

¶ Frozen formulation.

‖ Lyophilised formulation.

The AstraZeneca–Oxford University vaccine ChAdOx1 nCoV-19 (AZD1222) was the subject of a phase 1 clinical trial, for which the data have been published.^22^ The publication focused on the clinical reactions (ie, safety) of the vaccine and on the development of immune responses; the immunological data from day 28 and day 56 show the issue of comparability of assay results using different assay formats and target antigens. On day 28, using the in-house ELISA against S protein, the geometric mean titre (GMT) of the tested individuals was 157 ELISA units, with all participants showing signs of seroconversion. At the same timepoint, the GMT of sera in a 50% endpoint plaque reduction neutralisation titre of live SARS-CoV-2 was 218, with all participants showing signs of neutralising antibody. In a microneutralisation endpoint titre with an 80% endpoint, the GMT was 51, with 32 (91%) of 35 participants showing signs of seroconversion.^22^ At day 56, the GMT in the ELISA was 119 for those receiving a single dose and, in a separate laboratory that used total neutralisation as the endpoint, 23 (62%) of 37 participants receiving one dose had developed neutralising antibodies; all participants receiving two doses were seropositive and the median neutralising antibody titre was 29.^22^ The ELISA data derive from a single assay and are therefore comparable, with the results suggesting that the titre declined between day 29 and day 56 without a boost. The neutralisation assays on day 28 and day 56 were in a different format, so it is unclear whether there was a real decline. One way to clarify whether a decline in neutralising serum antibodies parallels the decline in ELISA antibodies would be to calibrate all assays against a common reference standard.

This approach would also be useful for comparing data from different trials. An adenovirus-vectored vaccine produced by CanSino Biologics (Tianjin, China) was used in a clinical trial at two dosage levels.^23^ In an ELISA using the receptor-binding domain as the target antigen, the vaccine produced a GMT of antibodies at day 28 of 656·5 units or 571·0 units in the two different dosing groups, with 244 (96%) of 253 individuals and 125 (97%) of 129 individuals exhibiting seroconversion.^23^ Neutralisation GMTs were 19·5 and 18·3 at the same timepoint, with seroconversion rates of 148 (59%) of 253 individuals and 61 (47%) of 129 individuals.^23^ The ELISA units and method are different from those of the Oxford trial^22^ and, although the format of the neutralisation assay is not given, it is almost certainly also different.

A third study involved the vaccine from the Gamaleya Institute in Russia.^24^ The vaccine has two components, one based on Adenovirus 26 and the other on Adenovirus 5. The Adenovirus 26 component is given first, followed by the Adenovirus 5 component 21 days later. Two presentations of the vaccine were tested, giving two groups of the study. After two doses, the ELISA GMTs were 14 703 and 11 143. The neutralisation titres were 49·25 and 45·95.

It is impossible to say whether these three vaccines induce similar responses. There is no reason why the results from these three studies should agree in numerical detail. The ELISAs are formatted differently with different antigens, and the output is in arbitrary in-house units. The formats of the neutralisation assays also differ, and the GMT and seroconversion rates cannot be compared with confidence. In this situation, the inclusion of a single reference antibody preparation would potentially make it possible to compare the different products and trials. In the absence of such a reference, it is clear that all three vaccines induce an immune response, but it is impossible to judge which, if any, is inducing higher antibody responses. Such a comparison would facilitate the defining of protective antibody titres and the possible establishment of efficacy and potency thresholds.

## Standardisation of immune response to COVID-19 by the International Standard

The WHO International Standard for anti-SARS-CoV-2 immunoglobulin facilitates comparison between clinical trials by expressing the results of neutralisation assays from different laboratories relative to the International Standard, which has an assigned potency in arbitrary units (IU). An arbitrary unit was chosen instead of a physical measurement (eg, ng of antibody) because polyclonal immune sera are complex mixtures of molecules with different specificities, but with a common biological effect (ie, neutralisation) which forms the basis for the range of relevant assay systems. Convalescent plasma or serum from patients are the preferred candidate standard because these samples most closely represent a clinical sample that is analysed in the assay. These samples are consistently able to reduce inter-assay variability when used as a calibrant for a range of tests, as in the case of many other viruses, including MERS-CoV.^25^, ^26^

**Figure 2:**
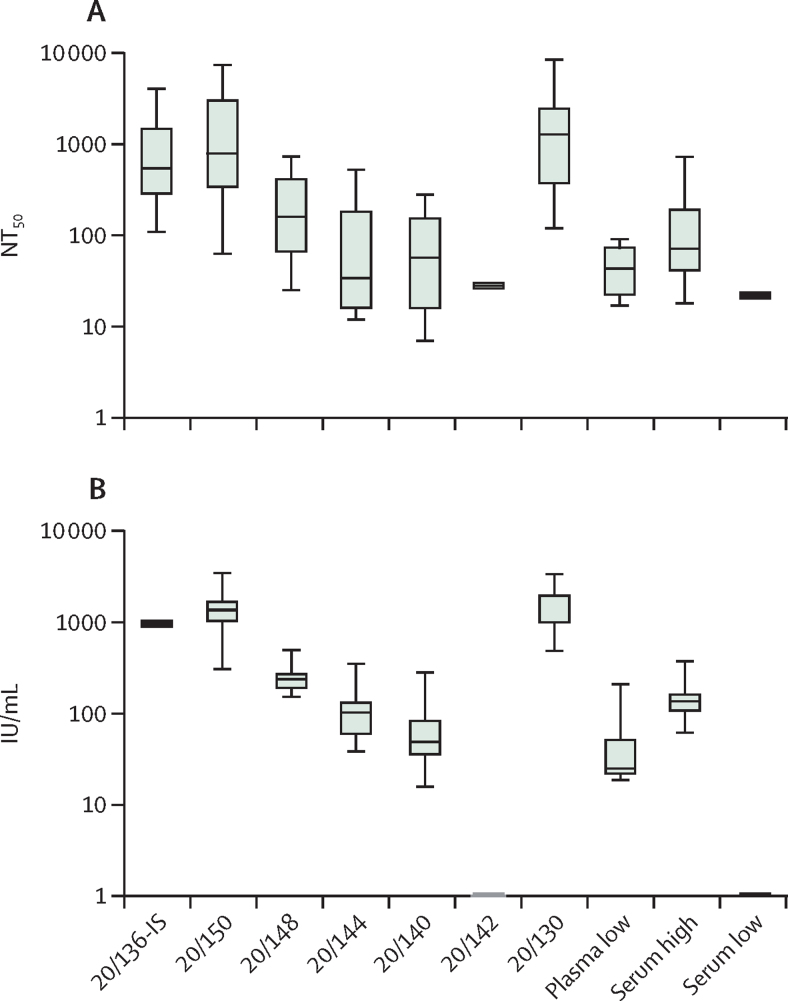
Harmonisation of live SARS-CoV-2 neutralisation assay results by the WHO International Standard (A) NT_50_ reported by participants.(B) Antibody potencies expressed as relative to the International Standard, 20/136 in IU/mL. The interquartile range of the values for each sample is represented as a box; the black line within the box marks the median; the bounds indicate minimum (lower bar) and maximum (upper bar) values. Further details on the codes for the WHO reference panel are published elsewhere.^9^ IU=International Units. NT_50_=50% neutralisation titre.

The WHO International Standard for SARS-CoV-2 immunoglobulin was characterised in an international collaborative study launched in July, 2020, which has since been completed;^6^, ^8^ 44 laboratories from 15 countries participated in the study using 125 methods, including 78 ELISAs, 27 neutralisation assays, and 20 other methods. The participants examined the candidate standard and a range of comparator samples, using assays routinely used in the group, and returned the data for central statistical analysis. The aim was to confirm that the standard is fit for purpose and to assess whether expressing data relative to the International Standard harmonises the data from various assays and samples. For example, the results of neutralisation assays with live virus or pseudotyped viruses can be compared directly or as titres relative to the reference. Figure 2A shows the spread of results obtained with the samples included in the study as provided by the participants; figure 2B shows the same data expressed as potency relative to the candidate reference. The spread in Figure 2B is much less than in figure 2A and the reference was established by the WHO Expert Committee on Biological Standardization partly on the basis of these findings.

The potency of the International Standard was assigned specifically for neutralisation assays, which have a clear functional biological readout. However, although the collaborative study results clearly showed less inter-laboratory variation when ELISA titres were reported relative to the International Standard, the WHO Expert Committee on Biological Standardization requested further data in order to assign an IU value to the International Standard for antigen-specific antibody binding assays, work which is ongoing. Meanwhile, the International Standard is available as a reference reagent for the harmonisation of related binding assays using an arbitrary binding antibody unit specific for each viral antigen (eg, the receptor-binding domain, S1, S, and nucleoprotein).^7^, ^8^

## Challenges and opportunities

The challenge in the production of high-order reference materials is that the time taken to produce them does not correspond to immediate needs.^27^ Solutions to this problem are the provision of less characterised standards, which might be bridged to the International Standard when they are produced, and the retrospective application of a conversion factor to the IU. For example, 3 months after WHO announced that COVID-19 constituted a Public Health Emergency of International Concern on Jan 30, 2020, a research reagent for anti-SARS-CoV-2 antibody (code 20/130) was made available through a collaboration between the National Institute for Biological Standards and Control and the Coalition for Epidemic Preparedness Innovations. The research reagent 20/130 was convalescent plasma from one recovered patient with a relatively high titre of anti-SARS-CoV-2 antibody. This research reagent offered a tool to compare assay results between different laboratories. Furthermore, 20/130 has been retrospectively calibrated to the International Standard as part of an international collaborative study (table 2) and can now be considered a secondary reagent; this approach allows the over 250 end users who received 20/130 to immediately convert their data into international standard unitage. The appropriate and correct application of the International Standard is important. After infection or immunisation, antibodies will be induced against all immunogenic epitopes. Thus, there will be antibodies with biological activity, such as neutralisation, and others which bind to other regions of the antigen and proteins but whose presence might not correlate with the neutralising activities. There might also be antibodies that neutralise but are not detected in binding assays; for instance, neutralising antibodies directed at regions outside of the receptor-binding domain will not be detected by ELISAs using the receptor-binding domain as the target antigen. It cannot, therefore, be assumed that the activity in one type of assay, such as neutralisation, strictly parallels another, such as binding in an ELISA. Even between binding assays, antibody titres against two viral proteins (eg, nucleoprotein and S protein) might not necessarily correlate. This factor could be particularly relevant in situations where diagnostic assays (eg, for confirmation of natural infection) are conflated with quantitative assays that measure only vaccine antibody titres. For example, it is inappropriate to assign a protective titre for vaccine efficacy in IU/mL when using an assay that is not measuring an antigen associated with protection. Such cases have arisen for measles and rubella serological testing,^28^ and have led to a misplaced lack of confidence in the use of the International Standard.^29^

**Table 2:** Calibration of research reagent 20/130 in International Standard unitage

	**Geometric mean titre**	**95% CI**	**Unit**
Neutralising antibody activity	1300	981–1719	IU/mL
Anti-receptor-binding domain IgG	502	382–660	BAU/mL
Anti-S1 IgG	588	398–870	BAU/mL
Anti-Spike IgG	476	418–542	BAU/mL
Anti-nucleoprotein IgG	747	214–2606	BAU/mL

The research reagent 20/130 has been calibrated to the First WHO International Standard for anti-SARS-CoV-2 immunoglobulin (NIBSC code 20/136) as part of a multicentre collaborative study. IU=International Units. BAU=binding antibody units.

## The way forward

Following the establishment of the International Standard, it is now important to ensure the correct use of the antibody standard in vaccine clinical trials to assist in the interpretation of results by providing the basis for expression of antibody titres in IU. This approach is an opportunity for exploring and possibly defining correlates of protection in IU/mL. The International Standard also permits data sets across a range of assays to be compared by reference to the IU. This comparison is especially important because many vaccine candidates are being developed; following approval of the first SARS-CoV-2 vaccines, non-inferiority clinical trial design should be considered. In these cases, the role of the International Standard would be particularly important in efforts to establish correlates of protection. In future, regulatory authorities might consider using immunological data to extend licensure indications to include additional groups (eg, different ages and immunocompromised people).

The contribution of cellular immunity, which acts together with antibodies in the immune response to SARS-CoV-2, must not be overlooked. Work on variant SARS-CoV-2 strains seems to suggest that protection against moderate and severe disease can be provided by cellular responses in the absence of neutralising antibodies (eg, escape variants), although infection per se by the new variant might be compromised. Preliminary data have shown that the International Standard for SARS-CoV-2 is still able to neutralise the new variants B.1.1.7, B.1.351, and P.1, although at higher concentrations than the early 2020 isolates. As new variants emerge, the ability of the International Standard to serve as a calibrant (ie, to be diluted to produce a standard curve) requires constant monitoring.

All vaccine developers should report antibody results from clinical trials relative to the WHO International Standard. Although early clinical trial results might have not been reported in IU/mL because of the urgency for submitting the data for fast regulatory approval, moving forward, all vaccine developers should now calibrate their assays against the International Standard and report results in IU. Regulatory authorities should assist by requiring that data submitted to them include reporting in IU. Other incentives could be considered and include requirements from sponsors of clinical trials and funders of the vaccine development. Ethics committees should also introduce such a requirement because comparability of results greatly adds to the public health value of data. Pharmacopeial monographs would also be helpful in emphasising the importance of reporting data in IU. As with compliance with clinical trial registration, a highly impactful requirement would be if medical journals required use of the IU for publication. We believe it is time to consider such policy levers to increase usage of IU as a unique measurement system for expressing results from clinical trials in the common language of the scientific community.

The comparability of results from different immunogenicity studies has long been considered an optional extra. Any risk–benefit assessment of the appropriate use of the WHO International Standard would confirm the need for a common language for communicating the message regarding the clinical performance of COVID-19 vaccines.

## Declaration of interests

We declare no competing interests.
